# EBV, HHV8 and HIV in B cell non Hodgkin lymphoma in Kampala, Uganda

**DOI:** 10.1186/1750-9378-5-12

**Published:** 2010-06-30

**Authors:** Lynnette K Tumwine, Jackson Orem, Patrick Kerchan, Wilson Byarugaba, Stefano A Pileri

**Affiliations:** 1 Department of Pathology, School of Biomedical Sciences, College of Health Sciences, Makerere University, P.O. Box 7072, Kampala, Uganda; 2 Unit of Hematopathology, Institute of Hematology and Clinical Oncology "L. & A. Seràgnoli", Bologna University School of Medicine, 40138 Bologna, Italy; 3 Uganda Cancer Institute, Mulago Hospital, P.0.Box 3935, Kampala, Uganda; 4 Kuluva Hospital, P.O Box 28, Arua, Uganda

## Abstract

**Background:**

B cell non Hodgkin lymphomas account for the majority of lymphomas in Uganda. The commonest is endemic Burkitt lymphoma, followed by diffuse large-B-cell lymphoma (DLBCL). There has been an increase in incidence of malignant lymphoma since the onset of the HIV/AIDS pandemic. However, the possible linkages of HHV8 and EBV to the condition of impaired immunity present in AIDS are still not yet very clearly understood.

**Objectives:**

1. To describe the prevalence of Epstein-Barr virus, Human Herpes virus 8 and Human Immunodeficiency Virus-1 in B cell non Hodgkin lymphoma biopsy specimens in Kampala, Uganda.

2. To describe the histopathology of non Hodgkin lymphoma by HIV serology test result in Kampala, Uganda

**Method:**

Tumour biopsies specimens from 119 patients with B cell non Hodgkin lymphoma were classified according to the WHO classification. Immunohistochemistry was used for detection of HHV8 and in situ hybridization with Epstein Barr virus encoded RNA (EBER) for EBV. Real time and nested PCR were used for the detection of HIV.

The patients from whom the 1991-2000 NHL biopsies had been taken did not have HIV serology results therefore 145 patients biopsies where serology results were available were used to describe the association of HIV with non Hodgkin lymphoma type during 2008-2009.

**Results:**

In this study, the majority (92%) of the Burkitt lymphomas and only 34.8% of the diffuse large B cell lymphomas were EBV positive. None of the precursor B lymphoblastic lymphomas or the mantle cell lymphomas showed EBV integration in the lymphoma cells.

None of the Burkitt lymphoma biopsies had HIV by PCR. Of the 121 non Hodgkin B cell lymphoma patients with HIV test results, 19% had HIV. However, only 1(0.04%) case of Burkitt lymphoma had HIV. All the tumours were HHV8 negative.

**Conclusions:**

The majority of the Burkitt lymphomas and two fifths of the diffuse large B cell lymphomas had EBV. All the tumours were HHV8 negative. Generally, the relationship of NHL and HIV was weaker than what has been reported from the developed countries. We discuss the role of these viruses in lymphomagenesis in light of current knowledge.

## Introduction

The frequency of non Hodgkin lymphomas (NHLs) has increased since the beginning of the HIV/AIDS pandemic in the early 1980s[1]. However, recently, studies have shown an association of non Hodgkin lymphomas (NHLs) with two gamma herpes viruses, Epstein-Barr virus (EBV) and Kaposi sarcoma herpes virus (KSHV)/Human herpes virus-8(HHV 8)[2,3].

EBV is important in the causation of Burkitt lymphoma (BL), Hodgkin lymphoma and other non Hodgkin lymphomas whereas HHV8 serves as an important

co-factor in the pathogenesis of primary effusion lymphoma (PEL) and large B cell lymphomas arising in patients with multicentric Castleman's disease who are profoundly immunosuppressed[4,5].

HIV associated lymphomas are high grade and of B cell origin. They are mainly extranodal and have poor prognosis.

They are broadly categorised as systemic lymphomas and primary central nervous system lymphomas. The systemic lymphomas include Burkitt lymphoma, diffuse large B cell lymphoma with immunoblastic (IB) morphology, HHV8+ primary effusion lymphoma (PEL) and its solid variant and plasmablastic lymphoma (PBL)[6].

In Western Europe and America, approximately 50-70% of HIV associated-DLBCL are EBV positive and express the transforming latent membrane protein-1(LMP-1). LMP-1 plays a critical role in the transformation of B lymphocytes. The effect of EBV LMP-1 in DLBCL is strongest in tumors representing a post germinal centre differentiation profile[7].

Since the majority of HIV -associated DLBCL are EBV positive, the defective T-cell immunity created by the HIV infection leads to promotion of EBV driven B cell proliferation hence lymphoma. However the disparities between EBV driven lymphoproliferations in transplant patients and those in HIV patients suggests that other factors are necessary for genetic damage in HIV associated-DLBCL[2].

HIV related BL in Western Europe and America have 30-60% EBV positivity. However, unlike DLBCL, the transforming EBV LMP-1 is not expressed in BL.

EBV supports BL tumour development. Epstein Barr nuclear antigen-1 (EBNA-1), a viral protein required for the replication and maintenance of the latent viral episomal DNA is consistently found in BL cells[7].

In vitro studies have shown that EBNA-1 transgenic mice develop B cell lymphoma with a very long latency, and EBNA-1 and c-myc may cooperate in its development[2,8].

The presence of latent EBV in B cells promotes genetic instability and therefore suggests that latent EBV could contribute to genetic alterations required for development of BL. In addition, some latent EBV transcription patterns found in BL produce viral proteins that are likely to protect BL cells from apoptosis induced by deregulated c-myc expression[9,10].

Given the strong apoptotic effects caused by over expression of c-myc, the role of EBV in some cases of BL is thought to be that of protecting BL cells from this side effect of c-myc translocation[11].

EBV-associated B cell lymphomas are caused by mutations and translocations of genes at various stages of differentiation and associated with expression of EBV latent genes. EBV latency 3 type antigens, such as the transforming latent membrane protein-1 and 2 and EBNA-2, have transformed cultured cells. In contrast, the majority of primary central nervous system lymphomas (PCNSL) are large cell, monoclonal B cell lymphomas that are clonally infected with EBV, although typically lacking the c-myc translocation[12].

The oncogenic potential of HHV8 in B cells is however less understood[13]. However HHV8 is an important factor in the oncogenesis of primary effusion lymphoma (PEL). Studies have shown that when B lymphocytes are infected by HHV8 in vitro, B cell transformation does not occur [14].

Cell lines have been derived from PEL specimens infected with HHV8 and they are being used for studying the molecular effects of HHV8 gene expression on B cells[15]. Genomic studies have shown that multiple copies (50-150 copies/cell) of episomal HHV8 genomes occur in PEL cells and when tested for presence of HHV8, all PEL cells were infected with HHV8 hence suggesting a role of HHV8 in PEL[15].

There is clonal expansion of the HHV8 infected cells with latent gene expression that causes neoplastic transformation through mechanisms of increased proliferation and impaired apoptosis. However, the true role of lytic genes during neoplastic transformation in vivo is not known[16].

In most cells, the latent viral gene expression pattern involves the expression of the latency associated nuclear antigen(LANA), a viral D-type cyclin homologue (vcyc), a viral homologue of FLICE inhibitory protein (vFLIP), a pre-miRNA transcript encoding 11 viral miRNAs, as well as vIRF3/K10.5/LANA-2. In addition a homologue of IL-6 is also expressed in some PEL cells[17].

Human immunodeficiency virus (HIV) is a lentivirus (a member of the retrovirus family). Historically, the retroviruses, have generally not been considered as causes of human cancer. The pathogenesis of AIDS-associated malignancies is thought to be the result of an opportunistic proliferation due to an oncogenic stimuli and a depressed immune system[18].

HIV is now thought to have a more direct transforming role than earlier thought as evidenced by studies done by McGrath in Kaposi sarcoma lesions where he found HIV-1 integrated in macrophages surrounding the early KS lesion but not in later more developed lesions[18,19].

The integrated provirus and the expressed HIV-1 gene products are thought to stimulate the surrounding macrophages to produce activating cytokines and hence cause proliferation[20].

A common integration site of HIV-1 is on the *c-fes/fps *oncogene. This is relevant to tumorigenesis because *c-fes/fps *oncogene encodes a protein-tyrosine kinase that has been implicated in controlling transformation of haemopoietic cells. The 92kD *c-fes/fps *protein signals through macrophage activating cytokines (IL-3, GM-CSF, and M-CSF). Integrated macrophages could lead to a complicated interaction with B-cells and T-cells via cell-cell signalling. Up regulation of *c-fes/fps *signalling has been shown in cells with the integrated HIV-1 provirus. These studies were however done in non B cell lymphomas but however prove that these lymphomas developed non -randomly[18,21].

HIV infected macrophages simulate an environment that is necessary for oncogenesis by up regulating growth factors, M-CSF, IL-8, IL-6, IL-10 and may become clonal. The paracrine effects lead to the surrounding B cells being stimulated into activation and proliferation[18,21].

In sub Saharan Africa, EBV, HHV8, HIV are endemic. This paper discusses the role of these viruses in lymphomagenesis in light of current knowledge.

## Methods

### Study design

A cross sectional descriptive design was used to describe the prevalence of Epstein Barr virus(EBV), Human Herpes virus 8(HHV8) and Human immunodeficiency virus-1(HIV-1) in the B cell non Hodgkin lymphoma biopsy specimens (1991-2000). We also described the histopathology of non Hodgkin lymphomas (2008-2009) by HIV serology test result. Routine HIV counselling and testing became available in 2004 which was well after the initial study period (1991-2000).

### Histopathology

Six hundred formalin fixed paraffin embedded tissue blocks with a diagnosis of non Hodgkin lymphoma were collected from the archive of the Department of Pathology, School of Biomedical Sciences, Makerere University College of Health Sciences from 1991-2000, 2008-2009. They were reassessed, stained with haematoxylin and eosin and Giemsa as previously described[22] Only 129 (1991-2000) were suitable for tissue micro array construction. The corresponding patient clinical case notes and histology report forms were also retrieved.

During 2008-2009, 142 biopsies from patients with known HIV serology test results were also analysed.

### Immunohistochemistry

Tissue micro arrays were constructed from the individual paraffin blocks and then subjected to monoclonal antibodies (CD3, CD5, CD10, CD20, CD30, CD38, CD79a, BCl-2, BCl-6, Ki-67, CD138, IRTA-1, MUM-1/IRF4, LANA-1) as previously described[22].

### In situ hybridization

EBV was detected by looking for the presence of EBV encoded RNA (EBER). It was assayed using in situ hybridisation with a FITC labelled probe to EBER-1 and 2 (Dako Y0017) and mouse anti-FITC (Dako M0878), rabbit anti-mouse serum and APAAP complexes as described previously[22]. Positive and negative controls were run concurrently.

### Polymerase chain reaction (PCR) for detection of HIV in formalin fixed paraffin embedded tissue blocks

DNA was extracted from the paraffin blocks as previously described [22]and Real time-PCR and nested PCR were used to detect the presence of HIV-1 [5], by looking for adequate preservation of the PLZF gene (300 bp).

#### a) PCR HIV-1 env NESTED

Amplification reactions were carried out in an automated thermocycler (mastercycler eppendorf) according to the following PCR protocol. Each PCR reaction included 100 ng of DNA, 25 μM of each specific primers (gene env of HIV-1), 0,2 mmol/L dNTP, PCR buffer 10× with MgCl _2 _(Invitrogen), MgCl _2 _25 mM and Taq pol. 5Uμ. The cycling parameters were as follows: One cycle of denaturation, annealing and extension were carried out at 94°C for 1 min, 62°C for 10 sec, 72°C for 10 sec respectively.

Secondly, 40 cycles of denaturation, annealing and extension were carried out at 94°C for 10 sec, 94°C for 10 sec, 94°C for 10 sec respectively. Two more cycles of extension were carried out at 72°C for 7 min and 4°C respectively.

The positive and negative controls were DNA extract from 8E5LAV cells and DNA extract from Jurkat respectively.

#### b) Real Time PCR protocol

The specific primers for gene fragment: gag HIV-1. The Real-Time reagent used was Quantitect SYBR Green PCR kit (Qiagen) according to the manufacturer's instructions.

Amplification reactions were done in LightCycler Instrument Roche software analysis: LightCycler 5.3.2. The positive and negative controls were DNA extract from 8E5LAV cells and DNA extract from Jurkat respectively.

The cycling parameters were as follows Taq Activation (Hot-Start) at 95°C for 15 min followed by 45 cycles of amplification for denaturation at 94°C for 10 sec, Annealing at 55°C for 30 sec, extension at 72°C for 30 sec and analysis temperature 78°C for 3 sec*

* To exclude unspecific products and dimer primers.

The probes used for these analyses explored the gag and env regions and spanned 142 and 248 base pairs respectively [6].

### Microscopy

Slides were mounted with a glass cover slip and analyzed with an Olympus BX61 microscope (Olympus, Tokyo, Japan).

### Data management and analysis

Data were collected and entered into the computer using EPI INFO software (supplied by CDC and WHO) for storage and initial analysis. Further analysis was done using SPSS software (SPSS, Chicago, IL)[23]. The data were summarized in frequency tables and graphs. For continuous variables such as age, the relevant measures of central tendency (means for normally distributed data and medians and interquartile ranges for skewed data) were used to explore the data. Fisher exact test was used for comparison of frequencies. The Mann-Whitney U test was used for unpaired comparison of continuous variables. A p value of less than .05 was considered significant[24].

### Ethical considerations

Permission to conduct the study and ethical clearance were obtained from the Research and Ethics committee of the Faculty of Medicine, Makerere University. A waiver of consent to use the patients' de linked HIV serology data was also obtained.

## Results

Of the 129 samples used for TMA construction, 10 cases (8%) were excluded from the study. Two were reclassified as lymphocyte depleted classic Hodgkin lymphoma and 2 others as anaplastic large cell lymphoma after immunohistochemistry. In the other 6 cases, the core biopsy was not representative enough of neoplastic tissue.

A total of 119 non Hodgkin B cell lymphoma biopsies were analyzed. According the 2001 WHO classification of haematopoietic and lymphoid neoplasms, they were classified as: 95 (79.8%) Burkitt lymphoma, 19 (16.0%) diffuse large B cell lymphoma, 4 (3.4%) mantle cell lymphoma and 1(0.84%) precursor B lymphoblastic lymphoma.

### Distribution of EBV by lymphoma type

Of the 119 B-non Hodgkin lymphomas, only 109 had EBV test results by in situ hybridisation,10 were not well preserved and internal controls could not be detected and were therefore excluded from the final analysis. Eighty seven (79.8%) cases tested positive for EBV and 22(20.2%) tested negative.

Of these, 86 were Burkitt lymphoma, of whom 79 (91.9%) tested EBV positive and 7 (8.1%) were EBV negative.

Among the diffuse large B cell lymphomas 18 had EBV test results. Eight (44.4%) were EBV positive and 10 (55.6%) EBV negative (Table 1).

**Table 1:** Distribution of non Hodgkin lymphoma types by Epstein Barr (EBER) test result, Makerere University, 1990-2000

Lymphoma type	EBER RESULT		Total
	Positive (%)	Negative (%)	
Burkitt lymphoma	79(91.9)	7(8.1)	86
Diffuse large B cell lymphoma	8(44.4)	10(55.6)	18
Precursor B lymphoblastic lymphoma	0(0.0)	1(100)	1
Mantle cell lymphoma	0(0.0)	4(100)	4
Total	87(79.8)	22 (20.2)	109

BL cases were more likely to test positive for EBV than the others, OR 4.9(95% CI 2.0 - 11.7) p < 0.01)

Burkitt lymphoma cases were more likely to have a positive EBV result: OR 4.9 (95% CI 2.0-11.7).

### Distribution of EBV by age and gender

**Figure 1:**
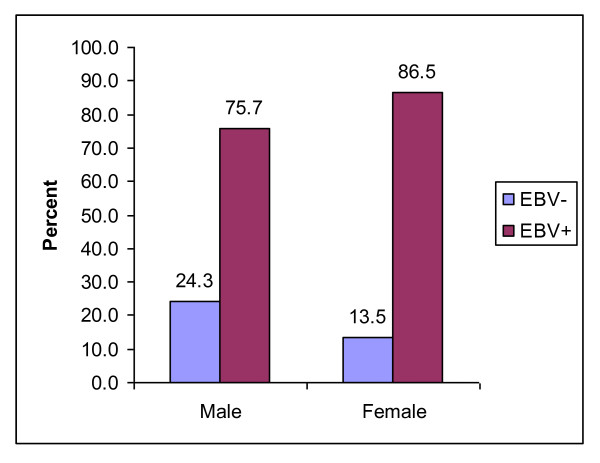
**Distribution of 107 B cell non Hodgkin lymphoma cases by gender and EBV test result, 1991-2000**.

Of the 105 non Hodgkin lymphomas with gender and EBV results, 70 (66.7%) were male and 35 (33.3%) were female. Seventeen (24.3%) of the males were EBV negative and 53 (75.7%) were EBV positive; 5 (13.5%) of the females were EBV negative and 32 (86.5%) were EBV positive. The difference was not statistically significant. (Figure 1)

**Figure 2:**
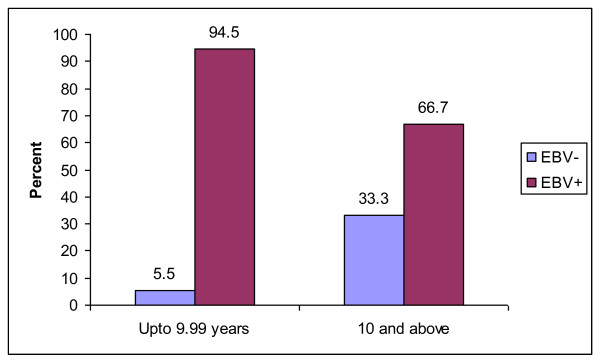
**Distribution of 107 B cell non Hodgkin lymphoma cases by age and EBV test result, 1991-2000**.

Of those that were aged less than ten years, 3 (5.5%) were EBV negative and 52 (94.5%) were EBV positive. In the more than 10 year age group 16 (33.3%) were EBV negative and 32 (66.7%) were EBV positive. The children below ten years of age were more likely to test positive for EBV and this was statistically significant OR 1.42 (95% CI 1.2-1.8), p = 0.001 (Figure 2)

### Distribution of EBV by anatomical site

As regards the distribution of EBV by primary anatomical site of disease, the following were EBV positive; all the 17 tumours arising in the abdomen, 7 (87.5%) of the 8 tumours arising in the jaw, 15 (60%) of the 25 tumours arising in the lymph nodes, 18 of the 19 (94.7%) gonad tumors, all the 3 tumours arising in the kidney and finally all six tumours with unspecified sites of origin.

### HHV 8 association with non Hodgkin B cell lymphoma

None of the cases studied had HHV 8.

### HIV association in Burkitt lymphoma biopsy samples

Since routine counselling and testing for HIV in lymphoma patients was not available during the initial study period we sought to find out if any of the Burkitt lymphoma biopsies had HIV. After extracting DNA from the formalin fixed paraffin embedded tissue blocks we used PCR to assess for presence of HIV. None of the 95 Burkitt lymphoma biopsies (1991-2000) showed HIV integration.

### HIV serology association with non Hodgkin B cell lymphoma

In order to evaluate the relationship between HIV and other non Hodgkin lymphomas, we prospectively analysed 145 lymphomas seen in the department of Pathology, Makerere University College of Health Sciences during the period 2008-2009 when routine HIV counselling and testing was available.

Of the 145 patients with non Hodgkin lymphoma, 121/145 (83%) had an HIV serology test result. Twenty three (19%) tested HIV positive, 98 tested negative.

According the 2001 WHO classification of haematopoietic and lymphoid neoplasms, they were classified as: 21/23 (91.3%) diffuse large B cell lymphoma, 1 Burkitt lymphoma and 1 small lymphocytic lymphoma. Most of the patients with DLBCLs were adults 17/21(81%) and had abdominal disease 11/21(52%).

## Discussion

Epstein Barr virus (EBV), human herpes virus 8(HHV 8) and the Human immunodeficiency virus (HIV) are part of the pathogenesis of a wide range of malignant lymphomas[2]. EBV is ubiquitous in the general human population and in Africa, children are infected quite early[25]. The prevalence of HHV 8 varies with geographical location and is high in the tropics[26,27].

The HIV pandemic in Africa has led to an increase in the incidence of tumors associated with these viruses, Kaposi sarcoma and non Hodgkin lymphoma[28].

Previous studies have shown high prevalence of these viruses in sub Saharan Africa. We sought to describe the prevalence of these viruses in biopsy specimens of Ugandan non Hodgkin lymphomas[29].

Only 121 non Hodgkin lymphomas were studied. Of these, 119 (98.3%) were B cell lymphomas and 2 (1.7%) were T cell lymphomas. This large majority of B cell lymphomas in our series is similar to what Cool and others found in their study in Kenya where the majority were B cell lymphomas[30].

The B cell lymphomas were classified as Burkitt lymphoma(95), diffuse large B cell lymphoma(19), mantle cell lymphoma(4) and precursor B lymphoblastic lymphoma(1) as we reported previously[22].

The prevalence of Epstein Barr virus (EBV) was very high among B cell lymphomas particularly Burkitt lymphoma. This is not surprising as it has been previously reported by Olweny and others [31-33]. However, the diffuse large B cell lymphomas (DLBCL) showed weaker EBV association of 44.4%. This is similar to what Cool found in neighbouring Kenya: 43% prevalence[30].

Children who were less than ten years of age were more likely to have EBV. Since most of the children had Burkitt lymphoma, this partly explains this stronger childhood EBV association in our study. Lazzi found that Kenyan children less than 15 years with Burkitt lymphoma had 94% EBV positivity[34]. Previous studies have also shown much earlier transmission of EBV in the African population as compared to the United States and Europe[35]. Geser and others also found that children that eventually developed Burkitt lymphoma had two times higher antibody titres to EBV as compared to those that did not[36].

There were no significant differences as regards gender and EBV association. This is not surprising since we know that EBV is acquired mainly horizontally by intimate contact[37]. More than 90% of the world's population carry EBV as a lifelong, latent infection of B lymphocytes[38].

As regards anatomical site of tumour presentation, all the tumors arising in the jaw, abdomen and kidney were EBV positive. These extranodal tumors were mainly endemic Burkitt lymphomas that are strongly associated with EBV[31]. However, there were no similar studies relating EBV status to anatomical site with which we could compare our findings. The nodal tumors had a weaker association with EBV-these were mainly diffuse large B cell lymphomas. This could be due to differences in the mechanisms responsible for oncogenesis in the different lymphoma types[2]. None of the tumors tested had HHV8. This is not surprising since an earlier study by Engels and colleges showed only one case of HHV8 positive lymphoma in Ugandan[39].

Using PCR, we did not find HIV in any of the Burkitt lymphoma including the Burkitt lymphoma plasmacytoid group that is associated with HIV in Western countries. Only one of the Burkitt lymphomas diagnosed in the period 2008-2009 had HIV. This is similar to what Parkin found in his case control study in children with Burkitt lymphoma[29].

Distinct pathways are involved in the molecular pathogenesis of HIV-related NHLs. EBV is an important etiological factor in a wide range of B cell lymphomas, however its strongest association is with endemic Burkitt lymphoma[7]. EBV associated B cell lymphomas arise as a result of mutations and gene translocations that occur at varying stages of differentiation. They express EBV latent genes. There are three types of latency antigens that have the ability to transform B cells, latent membrane protein 1 and 2(LMP-1 and 2) and Epstein Barr nuclear antigen-2 (EBNA-2)[18].

In DLBCL, LMP-1 is frequently expressed and plays a crucial role in the transformation of B cells. It activates the NFκβ, JNK, and p38 pathways by recruiting cellular TRAF 1-3 and TRADD molecules to 2 short sequence motifs CTAR-1 and CTAR-2 respectively, in the cytoplasmic domain of the LMP-1 molecule[2]. LMP-1 increases the expression of the antiapoptotic proteins A20 and bcl-2, the adherence molecule ICAM-1, the cell cycle regulator p27^Kip ^, and many others[40].

Evidence that the viral oncoprotein LMP-1 plays a role in lymphoma pathogenesis has arisen from the observation that knocked down LMP-1 cell lines of AIDS-DLBCL results in apoptosis. LMP-1 together with other factors lead to the genetic damage that all contribute to the pathogeneis of AIDS-DLBCL[41].

It seems that HHV8 and HIV have no role in the pathogenesis of endemic Burkitt lymphoma. Recent studies have shown that EBV is directly reactivated by P. falciparum antigens such as PfEMP1 during malaria infections. There is increased viral load leading to polyclonal B cell activation and enhanced B cell survival which leads to development of endemic BL in children living in malaria-endemic areas[42].

## Conclusion

The majority of the Burkitt lymphomas and only two fifths of the diffuse large B cell lymphomas had EBV. All the tumors were HHV8 negative. This study confirms the very strong EBV association in Burkitt lymphoma.

Overall, the association of NHL with HIV was weaker than what has been reported from other countries. Improved treatment strategies for better outcomes in HIV-associated B cell NHLs should be targeted towards patients with DLBCL rather than Burkitt lymphoma.

## Competing interests

The authors declare that they have no competing interests.

## Authors' contributions

LKT conceived the idea, collected data, analysed it and drafted the manuscript

JO contributed data and revised the manuscript

PK contributed data and revised the manuscript

WB participated in the design of the study and revised the manuscript

SAP participated in the design of the study, provided ancillary diagnostic tests and revised the manuscript
